# Correlation of Humoral Immune Response in Southern Bluefin Tuna, *T. maccoyii*, with Infection Stage of the Blood Fluke, *Cardicola forsteri*

**DOI:** 10.1371/journal.pone.0045742

**Published:** 2012-09-27

**Authors:** Nicole T. Kirchhoff, Melanie J. Leef, Victoria Valdenegro, Craig J. Hayward, Barbara F. Nowak

**Affiliations:** 1 National Centre for Marine Conservation and Resource Sustainability, University of Tasmania, Launceston, Tasmania, Australia; 2 SARDI Aquatic Sciences, Lincoln Marine Science Center, Port Lincoln, South Australia, Australia; Universitat de Barcelona, Spain

## Abstract

The blood fluke, *Cardicola forsteri*, is a prevalent infection in ranched southern bluefin tuna. This project aimed to define the timing and intensity of the various developmental stages of *C. forsteri* within southern bluefin tuna as well as to relate infection to host pathology and immune response. Archival samples from several cohorts of *T. maccoyii* sampled from 2008 to 2010 were used in this study. The prevalence and intensity of *C. forsteri* infection was described using heart flushes and histological examination. Humoral immune response, i.e. *C. forsteri* specific antibody, lysozyme activity, and alternative complement activity, was also described. Based on the validated and detailed *C. forsteri* infection timeline, relationships between infection events, physiological response, and diagnosis were proposed. Immune response developed concurrently with *C. forsteri* infection, with the majority of physiological response coinciding with commencing egg production. Further research is needed to confirm the origin of *C. forsteri* antigen which is responsible for immune response development and how *T. maccoyii* immune response works against infection. To aide this research, further diagnostic methods for confirmation of infection need to be developed.

## Introduction

Understanding the physiological effects and immune response to blood fluke infection is not only important in assessing its economic impact on the southern bluefin tuna, *Thunnus maccoyii*, industry, but also in developing treatments to reduce infections or, potentially, to prevent infections all together. Like other blood flukes, *Cardiola forsteri* has a two-host lifecycle, which requires a definitive host, bluefin tuna, *Thunnus* spp., and an intermediate host, a marine polychaete *Longicarpus modestus* (see [1]). Identified in ranched *T. maccoyii*, in 1997 [2], *C. forsteri* has been found to infect *T. maccoyii* off the coast of Australia [2]–[5] and Atlantic bluefin tuna *T. thunnus* off the United States of America [6], off the coast of Spain [7] and in the Adriatic Sea [8]. Prevalence and intensity of *C. forsteri* infection increase rapidly during ranching [9], with both the definitive and intermediate host present within the Tuna Offshore Farming Zone of Port Lincoln, South Australia [1]. Currently, the only pathological signs of infection have been limited to histological changes within the heart and gills [10], with no *C. forsteri* related pathology located within any other organs including kidney, spleen and liver [3]. No correlation has been found between infection and host condition, or physiological changes in plasma glucose, lactate, pH or osmolality [11]. A significant negative correlation with hemaoglobin concentration and positive correlation with humoral immune response, i.e. lysozyme, was reported in a rare case of hyperinfection [12]. However, these physiological effects may only be associated with hyperinfection, as these correlations were not found in a study which compared uninfected and infected *T. maccoyii* [13]. Furthermore, heavy infections of *C. forsteri* are rarely observed in ranched *T. maccoyii* [12], [14]. This is thought to be a consequence of host immune response [15]. A specific antibody response to *C. forsteri* in August 2005 was positively related to the mean abundance of adult flukes in May 2005 in fish in the same pontoon, suggesting a delay between the infection and antibody response [16]. Currently, we do not understand what triggers anti*Cardicola* antibody activity to develop and how it acts against *C. forsteri*.

Understanding the timing and intensity of the various developmental stages of *C. forsteri* within *T. maccoyii*, may allow further understanding of host response to infection. First, the current infection timeline, inferred by a stochastic model based on size and reproductive status of adult flukes and biological data inferred from other fluke species [17], needs to be validated. Although, *C. forsteri* eggs have been observed within the spongy layer of the ventricle [3], [10], in the afferent filamental arteries and in the lamellae of the gills [3], they have not been quantified nor the timing of their arrival within various organs has been described. The aims of this study were: (1.) to describe the timing of the appearance of the *C. forsteri* egg stage, (2.) to determine the timing of specific *C. forsteri* antibody activity development, and (3.) to correlate humoral immune response, i.e. lysozyme, alternative complement, and anti*Cardicola* antibody activity with the developmental stages of *C. forsteri* within *T. maccoyii*.

## Materials and Methods

### Ethics Statement

All work with animals, samples and methods for recovering samples were approved by the University of Tasmania board of animal ethics, project number A0010593.

### 2.1 Experimental Fish and Study Design

Archival samples from several cohorts of *T. maccoyii* sampled from 2008 to 2010 were used in this study (Table 1). Each tow of fish, which comprises of several schools of wild 2–4 year old *T. maccoyii*, is defined as a discreet cohort. Cohort_2009A_ and Cohort_2009B_ included samples which ranged from week 0, or at transfer into ranching pontoons, to harvest, at 19 weeks of ranching, and were used to assess long term effects as well as intra-annual variation. Cohort_2010_ contained weekly samples which ranged from 4 to 9 weeks of ranching and were used to more closely examine more closely the time period when 100% of *T. maccoyii* are expected to be infected and the adult flukes are expected to mature. Changes from the wild, i.e. largely uninfected *T. maccoyii*, were examined by comparing Cohort_wild_ to Cohort_2010_ as these samples were collected in the same season. As few, approximately 5–10% of wild *T. maccoyii* are believed to be infected [9]; it is assumed fish obtained directly from the wild would be previously uninfected. Inter-annual variation was examined by comparing Cohort_2009A_ and Cohort_2009B_ to Cohort_2010_. Due to low infection intensities of adult flukes in 2009 and 2010, additional archival samples were obtained from Cohort_2008_ which had comparatively higher mean intensity of adult *C. forsteri*. Unfortunately, histological samples were not available from Cohort_2008_; therefore these samples could not be used for correlations of immune response with presence of *C. forsteri* eggs.

**Table 1:** *T. maccoyii* cohort characteristics.

	Capture Date	Transfer Date	Stocking density(kg m^−3^)	length (cm)	weight (kg)	Sample type
Cohort_2008_	17.02.2008	20.03.2008	0.19–2.58	95.06	23.14	Cf_a_, B
Cohort_2009A_	19.03.2009	28.03.2009	n.a.	95.40	16.28	Cf_a_, Cf_eg_, B
Cohort_2009B_	23.03.2009	31.03.2009	n.a.	95.85	17.25	Cf_a_, Cf_eg_, B
Cohort_2010_	06.12.2009	26.12.2009	2.376	92.45	15.80	Cf_a_, Cf_eh_ Cf_eg_, B
Cohort_Wild_	21.01.2010	n.a.	n.a.	105.8	n.a.	Cf_a_, B

Several different types of samples were collected from each cohort: (Cf_a_) *C.forsteri* adult fluke count within the heart, (Cf_eg_ *C.forsteri* egg count within the gills, (Cf_eh_) *C.forsteri* egg and granuloma count within the heart, (B) blood serum. n.a. denotes data not available.

All cohorts of *T. maccoyii* were captured using purse seine in the Great Australian Bight. All *T. maccoyii*, with the exception of the Cohort_wild_ samples, were then towed to the Tuna Offshore Farming Zone of Port Lincoln, South Australia for commercial ranching. Cohort_wild_ was captured within the Great Australian Bight during the capture of wild fish for ranching and sampled prior to towing *T. maccoyii* back to Port Lincoln. The cohorts were then transferred into grow-out pontoons and fed a diet of domestic and imported baitfish for their entire ranching period, approximately 3 to 6 months.

### 2.2 Sample Collection

Each sampled *T. maccoyii* was captured using either a baited hook or by a commercial diver. Once landed on the boat, *T. maccoyii* were immediately spiked in the head, brain destroyed using a ‘Taniguchi tool’ (core) and a wire placed down the spine to destroy the upper spinal nerves. Total time between capture and killing of each *T. maccoyii* was less than 1 min. Whole blood was collected from the severed pectoral artery behind the pectoral recess in a 9 ml non-heparinized Vacutainer® tubes (BD, USA) and placed on ice. Blood was collected within 3 min of fish capture. The heart was placed in a waterproof tub, the visceral organs were placed in a waterproof bag and both stored on ice. The whole blood was used for serum collection and vials were stored upright at 4°C for 24 h, centrifuged at 1000×g at 4°C for 5 min, and serum aliquoted into three 1.5 ml tubes. Serum samples were stored at −20°C for up to one week and −80°C for long term storage.

Histological samples of the heart and gills were taken immediately upon returning to the laboratory, within 1–3 h of sample collection. Four 1 cm^3^ sections of heart were obtained from the apex of the ventricle, each section including both the compact and spongy layer. The heart samples were fixed in 10% neutral buffered formalin for 24 h. In Cohort_2010_ and Cohort_wild_, three sections of gill filaments were obtained from the second right gill arch: one at the each end and one from the centre of the gill arch. In Cohort_2009A_ and Cohort_2009B_, one section was obtained from the centre of the second right gill arch. Each section was excised from approximately halfway down the filament length. Histological examination from the middle of the second gill arch has been determined to be the most accurate for assessments of *C. forsteri* infection [18]. In Cohort_2010_ and Cohort_wild_, the gills were fixed in seawater Davidson’s fixative for 24 h. In Cohort_2009A_ and Cohort_2009B_, gills were fixed in 10% NBF for 24 h. All samples were then processed using standard histological techniques. After fixation, all tissues were cassetted and gills were decalcified for 1 h in Rapid Decalcifying Fluid (Australian Biostain, Australia). The heart and gill samples were then dehydrated in a graded ethanol series, cleared with xylene, embedded in paraffin, section at 5 µm and stained with haematoxylin and eosin. Histological sections of the gills were cut to maximize viewing of filaments and lamellae space where a majority of eggs were counted. This often came at the expense of viewing the filamental arteries, where a few eggs were observed.

### 2.3 Presence of C. forsteri

After histology sampling, hearts were dissected 2–4 h after removal from the carcass, and flushed with physiological saline to dislodge any adult *C. forsteri* (see [9]). Flushes were then poured into Petri dishes and examined for the presence of adults using a dissecting microscope. Cohort_wild_ hearts were stored frozen at −20°C. They were thawed and then flushed with physiological saline and flushes examined as described above. Number of adult flukes was reported as a total number per infected fish.

*C. forsteri* egg counts were completed on all four heart sections and at least 2 of the 3 gill sections, when available. The number of fish (n) used for histological examination are detailed in Table 2. All sections were viewed under a light microscope using a 40× objective and any eggs within the section quantified. Eggs were identified using a description provided in the literature [3]. An egg was counted if 100–50% of the internal integrity of the egg was intact. To describe the inflammatory response associated with eggs, in Cohort_2010_, eggs in the heart were further subdivided into those with or without an associated granuloma. An egg was considered one without a granuloma if there was little observed host response and 50–100% of internal integrity of the egg was intact. Eggs with granulomas were counted when a granuloma was visible, i.e. the egg was surrounded by a significant layer of host cells, and 20–100% of the internal integrity of the egg was intact. All histological slides were then scanned and analyzed with J-image version 1.45 to determine total surface area of the tissue. Egg counts were reported as number per cm^2^ surface area of histological section examined.

**Table 2:** Description of *C. forsteri* infection by % prevalence (P) (95% confidence interval) and mean intensity (I) (95% confidence interval) for adults within the heart, eggs within the heart and eggs within the gills of *T. maccoyii*.

Cohort	Ranching (week)	Adult Flukes			eggs cm^−2^ Heart			eggs cm^−2^ Gills		
		n	P	I	n	P	I	N	P	I
2009A	0 (transfer)	20	0.0 (0.00–37.71)^c^	0.00 (n.a.)	16	31.3 (13.22–56.38)^b^	6.60 (5.00–9.00)^b^	19	0.0(0.0–17.55)	0.00 (n.a.)
	8	20	70.0 (47.46–84.04)^b^	4.07 (2.21–9.64)	20	15.0 (4.22–37.22)^b^	2.67 (2.00–3.00)^c^	20	0.5 (0.26–24.42)	1.00 (n.a.)
	19	19	100.0 (83.32–100.0)^a^	4.8 (2.9–7.9)	20	100.0 (83.32–100.0)^a^	120.40 (92.15–164.35)^a^	20	25.0 (10.41–47.45)	90.4 (29.2–201.2)
2009B	0 (transfer)	20	0.0 (0.0–16.68)^c^	0.00 (n.a.)	20	35.0 (16.69–57.64)^b^	5.00 (2.86–9.43)^b^	19	0.0 (0.0–17.55)^b^	0.00 (n.a.)
	8	20	45.0 (24.43–68.0)^b^	3.33 (2.00–6.44)	20	50.0 (29.28–70.72)^ab^	6.9 (4.40–11.10)^b^	10	0.0 (0.0–29.08)^ab^	0.00 (n.a.)
	19	20	95.0 (75.58–99.74)^a^	7.74 (5.32–11.84)	20	100.0 (83.32–100.0)^a^	54.2 (37.90–79.00)^a^	20	30.0 (13.96–53.54)^a^	82.17 (22.00–196.50)
Wild	–	22	0.0 (0.0–15.7)^b^	0.00 (n.a.)^n.a.^	22	0.45 (0.24–22.21)^b^	4.00 (n.a.)^n.a.^	22	0.91 (0.16–29.07)^c^	3.00 (1.00–3.00)^b^
2010	4	8	87.5 (50.0–99.36)^a^	3.14 (1.86–4.71)^ab^	8	37.5 (11.12–71.07)^b^	11.33 (9.00–12.67)^a^	8	0.0 (0.0–36.46)^c^	0.00 (n.a.)^n.a.^
	5	6	100.0 (58.9–100.0)^a^	4.40 (2.40–6.00)^a^	6	66.7 (27.14–93.17)^a^	6.50 (5.00–8.75)^b^	6	83.3 (41.14–99.14)^ab^	69.80 (1.80–194.2)^ab^
	6	2	50.0 (2.5–97.5)^a^	2.50 (2.00–2.50)^ab^	2	50.0 (2.54–97.46)^ab^	20.00 (n.a.)^n.a.^	2	0.0 (0.0–77.63)^abc^	0.00 (n.a.)^n.a.^
	7	10	90.0 (55.36–99.48)^a^	2.33 (1.56–3.00)^ab^	10	100 (70.92–100)^a^	23.10 (13.00–35.30)^a^	9	100.0 (67.67–100)^a^	329.00 (141.11–582.00)^a^
	8	10	70.0 (38.1–91.3)^a^	2.6 (1.60–4.00)^ab^	10	100 (70.92–100)^a^	9.60 (5.10–17.10)^ab^	10	20.0 (3.68–55.35)^bc^	8.00 (1.00–15.00)^b^
	9	10	40.0 (15.01–70.91)^a^	1.50 (1.00–1.75)^b^	10	50.0 (22.25–77.75)^a^	3.60 (1.20–5.80)^b^	10	0.0 (0.0–29.08)^c^	0.00 (n.a.)^n.a.^

Significant letters denote statistical differences at p<0.05 within Cohort_2009A_, Cohort_2009B_ and between Cohorts_wild_ & Cohort_2010_. Intensity could not be directly compared statistically on samples with no infection present or with only one infected individual, denoted with a superscript n.a.

### 2.4 Anti*Cardicola* Antibody Activity

Anti*Cardicola* antibody titre was measured [16]. Optimization of the method using checkerboard analysis required changes to the concentration and identity of reagents used in the ELISA and Western blot: an increase in blocker concentration from 0.3% to 1% casein-TBS, a decrease in rabbit anti-tuna heavy chain immunoglobulin (RATH) antibody concentration from 1∶100 to 1∶200, and a change from sheep anti-rabbit IgG alkaline phosphatase conjugate (1∶8000, Sigma) to goat anti-rabbit IgG alkaline phosphatase conjugate (1∶2000, Sigma). Western blotting was used to investigate seropositive and seronegative samples [16]. Different seropositive and seronegative samples were used as there was no access to the control samples from the previous study [16]. The seropositive and seronegative controls were run on each plate. The dilution of each sample and control was converted into volume of serum within each well, and absorbance plotted against the log of serum volume. Titre, stated as antibody activity per volume of serum (units µl^−1^) was calculated [19]. A sample was defined as positive for anti*Cardicola* antibody activity when titre >1 unit µl^−1^.

**Figure 1:**
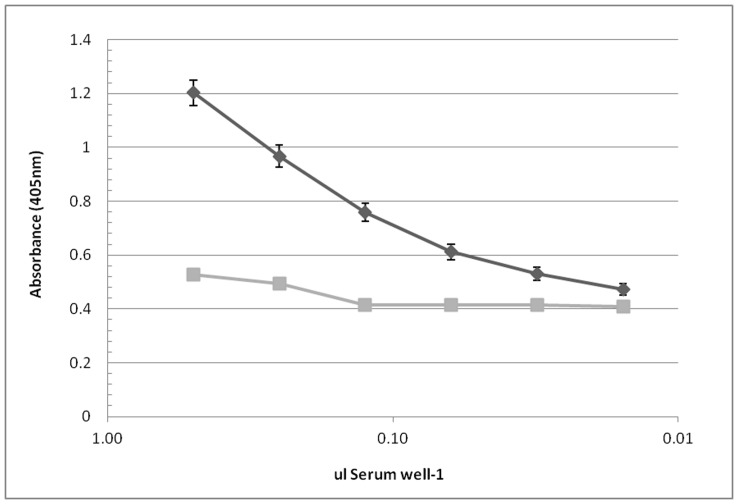
Anti*Cardicola* ELISA of positive (♦) and negative (▪) controls of *T. maccoyii* serum; mean ± SE of 15 observations.

The anti*Cardicola* antibody ELISA titration curve for the seropositive and seronegative controls was linear across the dilution range used in this study (Figure 1). At a 1∶400 dilution, or 0.13 µl serum well^−1^, the OD of the positive standard was 0.760±0.033 and the OD of the negative standard was 0.416±0.016. When each step was left out in sequence, background OD was <0.300 for 5 different plate observations.

### 2.5 Humoral Immune Response

Blood serum was analyzed in triplicate for lysozyme activity and alternative complement activity. Lysozyme activity was measured [20] using a modified method [21]. Blood serum alternative complement activity was measured [22] using a modified method [21].

### 2.6 Statistics

Description of *C. forsteri* infection stages and humoral immune response as well as their correlations were interpreted using the R2.12.2 statistical package (©2011, The R Foundation for Statistical Computing). Numerous comparisons were also made between cohorts, as detailed in section 2.1. Each stage of *C. forsteri* infection was described by prevalence and intensity [23]. Anti*Cardicola* antibody activity was described by prevalence (the number of hosts positive for immunoglobulin activity as a proportion of the total population). The prevalence was determined using a Sterne’s exact method at N = 1000±95% confidence interval. Fisher’s exact test was used to compare prevalence between cohorts and between sampling times. Mean intensity of *C. forsteri* infection was determined by bootstrapping at N = 2000±95% confidence interval. To examine changes in infection intensity and anti*Cardicola* antibody activity, Kruskal Wallis, or Mann-Whitney-Wilcoxon test for pairwise comparisons, was used with a Bonferroni p-values correction. For two-variable comparisons between cohorts and time, *C. forsteri* intensity and anti*Cardicola* antibody activity had to be rank transformed prior to using a two-way ANOVA with Tukey’s HSD post-hoc test. Humoral immune response was described using a two-way ANOVA followed by Tukey’s HSD post-hoc test. Assumptions were checked by the residual plot, with lysozyme activity arcsine transformed and anti*Cardicola* antibody activity log_10_+1 transformed for all statistical analysis due to their failure to conform to homogeneity of variances. Spearman’s two-sided correlation was used to compare humoral response to *C. forsteri* infection intensities at each ranching duration individually, at pooled ranching durations within each Cohort, and between Cohort’s. To further investigate the correlation between infection and antiCardicola activity within the hyperinfected Cohort_2008_, each individual activity value was adjusted by subtracting the value of the mean activity level for the corresponding week of ranching, thereby removing the effect of ranching duration. Significance for all statistical analysis was assumed at p≤0.05.

**Figure 2:**
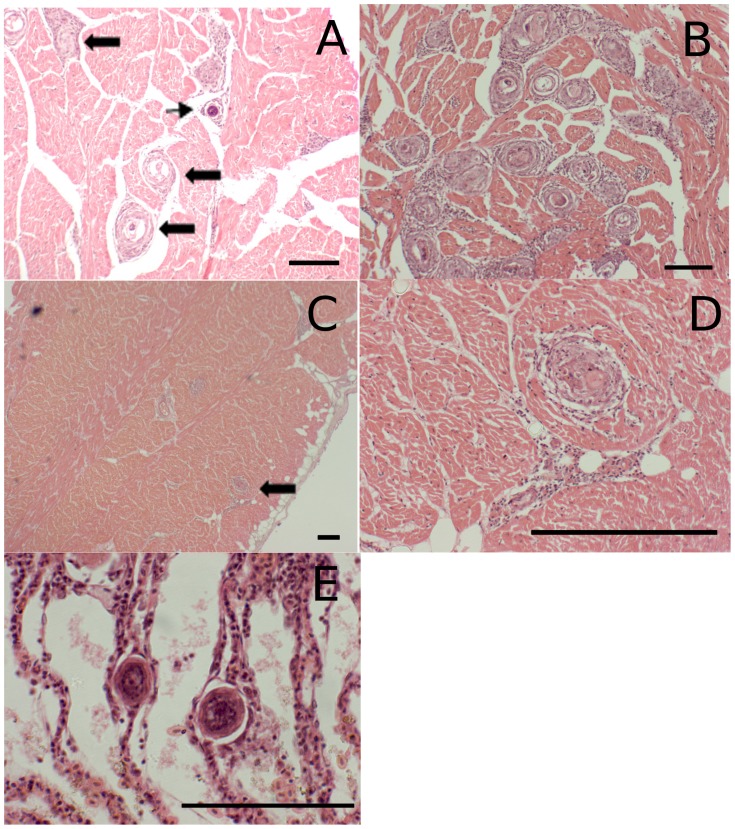
Presence of *C. forsteri* eggs in *T. maccoyii* organs H&E staining (A) within the spongiosa of the heart ventricles, eggs (small arrow) and granulomas (large arrow); (B) large number of granulomas associated with eggs within the spongiosa of the heart; (C & D) granuloma associated with eggs in the compacta of the heart ventricles (large arrow); (E) eggs within the lamellae creating a ‘string of pearls’ appearance. Scale bar 100µm.

**Table 3:** Presence of *C. forsteri* eggs in the heart of *T. maccoyii* with and without associated granulomas, % prevalence (P) (95% confidence interval) and mean intensity (I) (95% confidence interval).

		Eggs without granulomas		Eggs with granulomas	
Ranching(week)	n	P	I	P	I
4	8	25.0 (4.64–63.53)^b^	1.50 (1.00–1.50)^c^	37.5 (11.12–71.07)^b^	12.33 (11.00–13.00)^c^
5	6	50.0 (15.32–84.68)^ab^	2.33 (1.00–3.33)^bc^	66.7 (27.14–93.71)^ab^	8.5 (7.25–9.25)^d^
6	2	50.0 (2.54–97.46)^ab^	2.00 (n.a.)^n.a.^	50.0 (2.54–97.46)^ab^	22.0 (n.a.)^n.a.^
7	10	70.0 (38.06–91.27)^ab^	5.14 (3.29–8.00)^b^	100 (70.92–100)^a^	26.70 (17.80–40.00)^b^
8	10	100 (70.92–100)^a^	85.2 (53.30–140.90)^a^	100 (70.92–100)^a^	94.80 (60.90–160.50)^a^
9	10	100 (70.92–100)^a^	123.50 (85.00–152.60)^a^	100 (70.92–100)^a^	125.40 (86.70–154.20)^a^

Significant letters denote statistical differences at p<0.05. Intensity could not be directly compared statistically on samples with no infection present or with only one infected individual, denoted with a superscript n.a.

## Results

### 3.1 Characterization of C. forsteri Infection

All life stages of *C. forsteri* within *T. maccoyii* increased in prevalence and intensity during ranching (Table 2). Adult *C. forsteri* infection was significantly greater during ranching compared to wild *T. maccoyii*, with both prevalence and intensity peaking several times within the traditional ranching duration (Table 2). The prevalence of adult *C. forsteri* increased from 0% in Cohort_wild_ to 100% at week 5 of Cohort_2010_ (p<0.001), with Cohort_2010_ having higher prevalence than Cohort_wild_ at all sampling time points (p = 0.003). Both Cohort_2009A_ and Cohort_2009B_ increased from 0% prevalence at week 0, or the initiation of ranching, to ∼100% at week 19, or harvest (p<0.001). The mean intensity of adult *C. forsteri* increased 4× from Cohort_wild_ to week 5 of ranching in Cohort_2010_. Within Cohort_2010_, the intensity of infection decreased (Χ^2^ = 12.1327, df = 5, p = 0.033) from 4.4 at week 5 to 1.5 at week 9. In Cohort_2009A_ and Cohort_2009B_ the mean intensity of adult *C. forsteri* increased approximately 4× from week 0 to week 8 & 19 of ranching (Cohort_2009A_ X^2^ = 40.4442, df = 2, p<0.001; Cohort_2009B_ X^2^ = 36.8906, df = 2, p<0.001). There was a significant amount of intra-annual variation in the prevalence of adult *C. forsteri* (week 8: Cohort_2009A_ vs. Cohort_2009B_, p = 0.048), although no differences were found for mean intensity. There was no evidence of inter-annual variation in prevalence or intensity of adult *C. forsteri*.

**Figure 3:**
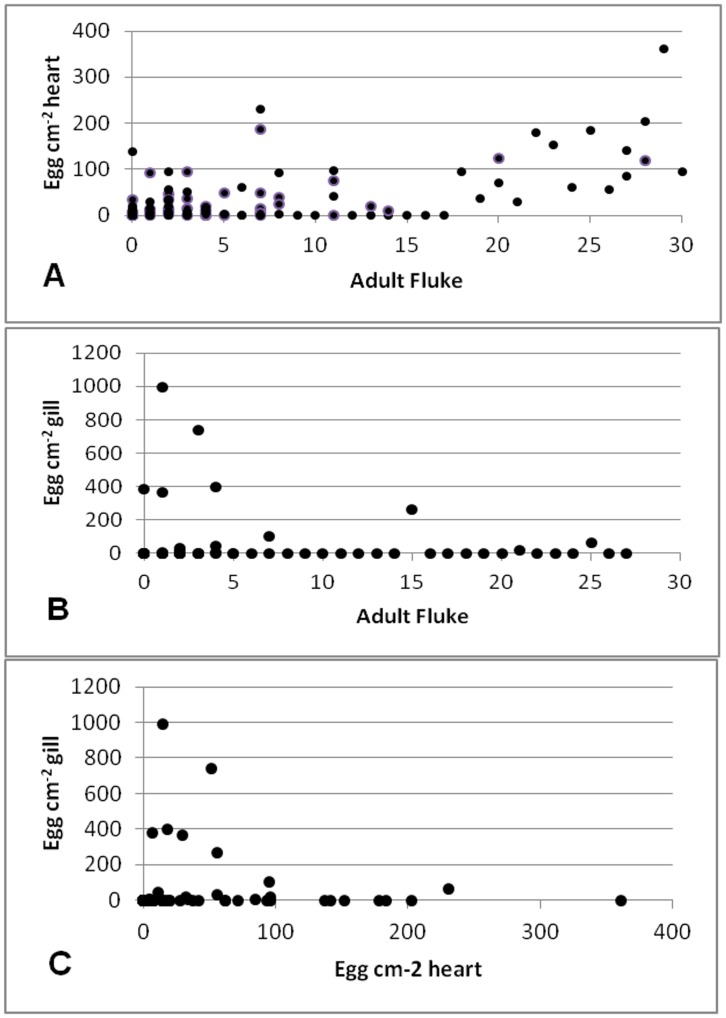
Correlation between (A) the number of adults and eggs in the heart, Spearman’s rho = 0.550, df = 184, p<0.001; (B) the number of adults and eggs in the gills, Spearman’s rho = −0.283, df = 175, p<0.001; (C) the number of eggs in the heart and eggs in the gills, Spearman’s rho = −0.387, df = 170, p<0.001 for Cohort_2009A_, Cohort_2009B_, and Cohort_2010_ combined.

The prevalence and intensity of *C. forsteri* eggs within the heart increased significantly during ranching, also peaking numerous times within the traditional ranching duration (Table 2). *C. forsteri* eggs were primarily found within the spongiosa of the heart ventricles (Figure 2A&B), with only a few eggs being found in the compacta (Figure 2C&D). Eggs within the heart were observed with or without granulomas and at various stages of break-down. No miracidium development was observed within the heart. In gills, eggs were observed at the tips of the lamellae, with only a few present within the lamellae (Figure 2E). Eggs were observed at various stages of development within the gills, some with fully developed miracidia. No host response, such as granuloma, was observed in association with eggs within the filaments or at the tips of the lamellae. The prevalence of *C. forsteri* eggs within the heart increased from 0.45% in Cohort_wild_ to 100% in Cohort_2010_ by 7 weeks of ranching (p<0.05). In Cohort_2009A_ & Cohort_2009B_, the prevalence of eggs within the heart increased from ∼31–35% at week 0 to 100% at week 19 (p<0.001). The mean intensity of eggs within the heart increased ∼6× from the wild to week 7 of ranching (χ^2^ = 5.8578, df = 0.016). Mean intensity of eggs within the heart increased from week 8 to week 19 in both Cohort_2009A_ and Cohort_2009B_ (Cohort_2009A_ X^2^ = 43.8447, df = 2, p<0.001; Cohort_2009B_ X^2^ = 39.6804, df = 2, p<0.001). In Cohort_2009A_, mean intensity of eggs within the heart decreased from week 0 to week 8, in contrast to Cohort_2009B_ which did not change. In Cohort_2010_, the prevalence of egg associated granulomas in the heart increased at the same rate as the prevalence of eggs without associated granulomas, so that at any given time point approximately 50% or more of the eggs were associated with granulomas. The mean intensity of granulomas associated with *C. forsteri* eggs within the heart increased approximately 2x× from week 4 and 5 of ranching to week 7, and an additional 5× from week 7 to week 8 and 9 of ranching (X^2^ = 34.658, df = 5, p<0.001) (Table 3). At harvest, a majority, if not all of the eggs in the heart were associated with granulomas in Cohort_2009A_ and Cohort_2009B_, although the eggs with and without granulomas were not quantified in the hearts of harvest fish due to time constraints. The maximum number of eggs found within the heart ventricle was 361.24 eggs cm^−2^ from Cohort_2009A_ at harvest, or 19 weeks of ranching. There was significant intra-annual variation between cohorts in the prevalence and mean intensity of eggs within the heart (week 8: Cohort_2009A_ vs. Cohort_2009B_ prevalence p = 0.041 intensity χ^2^ = 6.5926 df = 1 p = 0.010; week 19: Cohort_2009A_ vs. Cohort_2009B_ intensity χ^2^ = 10.5366 df = 1 p = 0.001). There was significant inter-annual variation between Cohort_2009A_ and Cohort_2009B_ and Cohort_2010_, with the prevalence of infection occurring approximately 1–2 weeks earlier in 2010 compared to 2009 (Cohort_2010_ at week 8 vs Cohort_2009A_ (p<0.001) and Cohort_2009B_ (p = 0.01) at week 8; prevalence Cohort_2010_ at week 7 vs Cohort_2009A_ (p<0.001) and Cohort_2009B_ (p = 0.01) at week 8; Cohort_2010_ at week 9 vs Cohort_2009A_ (p<0.078) and Cohort_2009B_ (p = 1.00) at week 8) There was no inter-annual variation in the mean intensity of eggs within the heart (p>0.01).

**Table 4:** Anti*Cardicola* antibody activity in *T. maccoyii* serum by prevalence (95% confidence interval) and mean activity ± SE.

Cohort	Ranching (week)	n	anti*Cardicola* Prevalence	anti*Cardicola* antibody activity units µl^−1^
2009A	0 (transfer)	20	70.0 (47.46–86.04)^ab^	19.132±7.886^a^
	8	20	55.0 (32.00–75.57)^b^	9.122±3.640^a^
	19	20	100 (83.32–100.00)^a^	72.160±11.095^b^
2009B	0 (transfer)	20	40.0 (20.90–62.77)^b^	4.078±1.570^a^
	8	20	95.0 (75.58–99.74)^a^	72.618±19.551^b^
	19	20	65.0 (42.36–83.31)^ab^	23.364±6.005^a^
Wild	–	22	31.8 (15.18–54.65)^b^	5.149±2.902^c^
2010	4	8	87.5 (50.00–99.36)^ab^	64.833±11.457^ab^
	5	4	75.0 (24.87–98.2)^ab^	86.978±37.078^ab^
	6	2	100 (22.37–100)^ab^	222.817±19.571^a^
	7	10	100 (70.92–100)^a^	99.327±16.474^ab^
	8	9	88.9 (55.66–99.43)^a^	81.460±32.010^ab^
	9	9	88.9 (55.69–99.43)^a^	34.918±17.049^b^

Significant letters denote statistical differences at p<0.05 within Cohort_2009A_, Cohort_2009B_ and between Cohorts_wild_ & Cohort_2010_.

The prevalence and intensity of *C. forsteri* eggs within the gills increased significantly during ranching yet were patchy in their distribution and presence (Table 2). The prevalence of *C. forsteri* eggs in the gills increased from 0.91% in Cohort_wild_ to 100% in Cohort_2010_ at week 7, but then decreased to 0% at week 9 of ranching. There was no change in the prevalence of eggs within the gills in Cohort_2009A_ over the ranching duration, with less than 30% of *T. maccoyii* positive with eggs in the gills at any sampling point, yet in Cohort_2009B_ egg prevalence decreased from week 0 to week 19 (p<0.01). The mean intensity of infection increased ∼100× between Cohort_wild_ and week 7 of Cohort_2010_ (X^2^ = 5.6561, df = 1, p = 0.017) but then decreased back to an intensity not statistically different from the wild by week 8. While the decrease in Cohort_2010_ between weeks 7 and 9 in the prevalence and intensity of eggs within the gills appeared to be gradual, there was no statistically significant difference between week 8 and 9. In Cohort_2009A_ and Cohort_2009B_, eggs were only found within the gills of *T. maccoyii* at week 19 of ranching at approximately 90 eggs cm^−2^ gill per infected *T. maccoyii*. The maximum infection intensity was 741 eggs cm^−2^ within Cohort_2010_ at 7 weeks. There was no evidence of intra-annual or inter-annual variation in the prevalence and mean intensity of *C. forsteri* eggs within the gills. There was a positive correlation between the number of adult flukes and eggs within the heart (Spearman’s rho = 0.550, df = 184, p<0.001) (Figure 3A), and a negative correlation between the number of adult flukes and eggs within the gills (Spearman’s rho = −0.283, df = 175, p<0.001) (Figure 3B) and between the number of eggs within the heart and gills (Spearman’s rho = −0.387, df = 170, p<0.001) (Figure 3C) in Cohorts_2009A_, Cohort_2009B_, and Cohort_2010_ combined. There two instances when eggs were observed but no adult fluke present, this may be due to the death and removal of the adult fluke prior to destruction and removal of the egg or a false negative of the diagnostic test.

**Table 5:** Humoral immune response (mean ± SE or SD) in *T. maccoyii*.

Cohort	Ranching(week)	n	Lysozyme Activity (µg ml^−1^)	n	ACH50(units ml^−1^)
2009A	0 (transfer)	20	25.43±8.99	20	156.40±18.63^a^
	8	20	171.88±17.34	20	115.20±12.30^b^
	19	20	33.49±4.81	20	48.20±4.10^c^
2009B	0 (transfer)	0	NA	0	NA
	8	20	83.74±8.21	20	114.20±7.37^a^
	19	20	96.08±14.05	20	57.10±6.70^b^
Wild	–	22	9.366±2.071^c^	22	167.337±16.626^b^
2010	4	8	144.143±29.156^b^	8	241.918±0.338^ab^
	5	4	295.975±63.698^a^	4	330.0589±0.526^a^
	6	2	198.200±7.200^ab^	0	161.401±41.028^ab^
	7	10	129.750±34.779^b^	9	212.658±21.926^ab^
	8	10	135.230±33.057^b^	8	157.976±29.173^b^
	9	10	110.490±30.230^b^	10	150.481±18.301^b^

Significant letters denote statistical differences at p<0.05 within Cohort_2009A_, Cohort_2009B_ and between Cohorts_wild_ & Cohort_2010_.

### 3.2 Development of Anti*Cardicola* Antibody Activity

Anti*Cardicola* antibody prevalence and activity increased over ranching duration (Table 4). Anti*Cardicola* antibody activity was present in 31.8% of Cohort_wild_ and increased to 100% by week 6 of ranching in Cohort_2010_. At the start of ranching, week 0, 70% of Cohort_2009A_ and 40% of Cohort_2009B_, were positive for anti*Cardicola* antibody activity. In Cohort_2009A_, the prevalence of anti*Cardicola* antibody activity increased 2× from week 8 to week 19. In Cohort_2009B_, the prevalence of anti*Cardicola* antibody activity increased 2× from week 0 to week 8. At week 6, anti*Cardicola* antibody activity was ∼6–44× higher in Cohort_2010_ compared to Cohort_wild_, (W = 160.5, p<0.001). Anti*Cardicola* antibody activity peaked in Cohort_2010_ at week 6 of ranching, decreasing by week 9 of ranching to an activity level still significantly higher compared to the wild (W = 166.5, p = 0.002). In Cohort_2009A_, anti*Cardicola* antibody activity increased 3.7× between week 0 and week 19 of ranching (X2 = 24.51, df = 2, p<0.001). In Cohort_2009B_, anti*Cardicola* antibody activity increased 18× from the commencement of ranching to week 8 (X^2^ = 21.0711, df = 2, p<0.001). There was a significant intra-annual variation in anti*Cardicola* antibody activity over ranching duration when Cohort_2009A_ was compared to Cohort_2009B_ (F = 22.4486, df = 2,144, p<0.001). There was significant inter-annual variation when Cohort_2010_ was compared to Cohort_2009A_ (p<0.01), but not when Cohort_2010_ was compared to Cohort_2009B_ (p>0.05) at week 8 of ranching. Therefore, it may be that the intra-annual variation in Cohorts is the most significant source of variation in anti*Cardicola* antibody activity.

**Figure 4:**
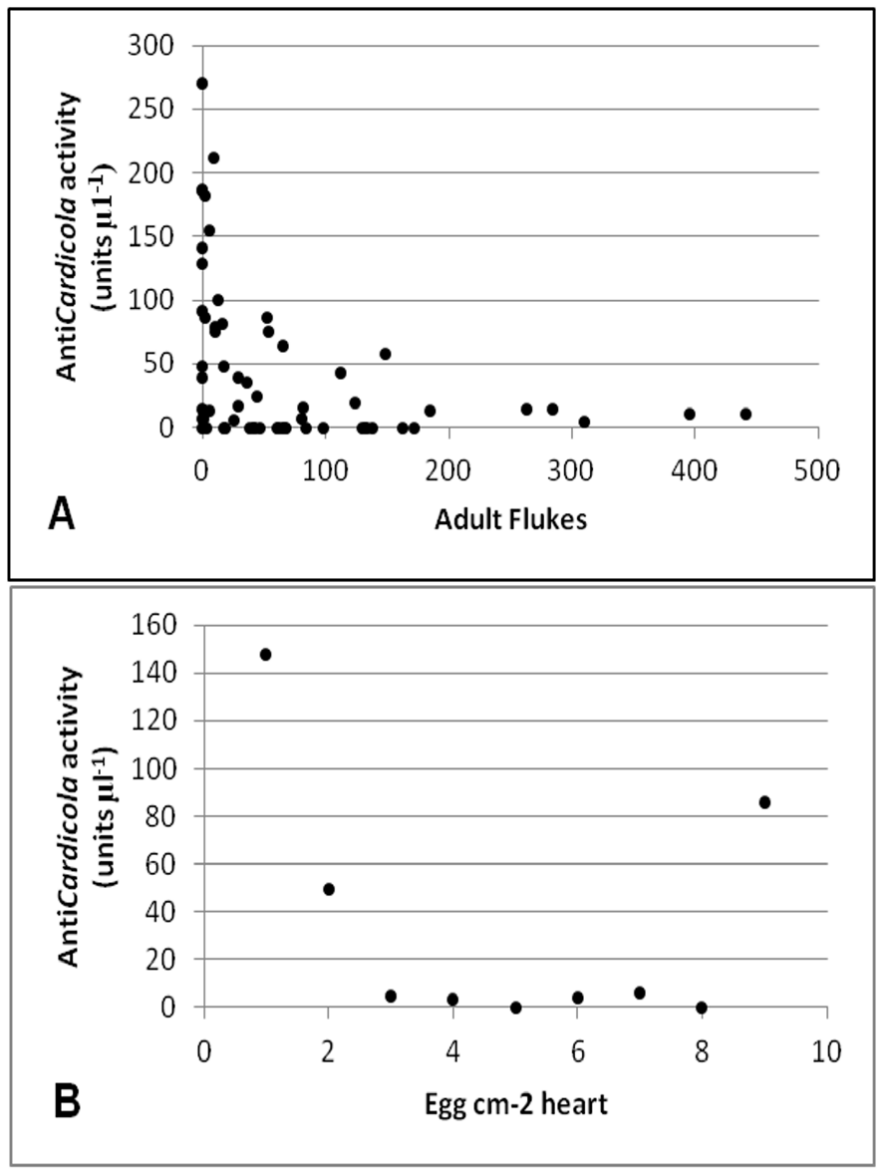
Correlations between Anti*Cardicola* activity in *T. maccoyii* serum and *C. forsteri*: (A) Cohort_2008_, Spearman’s rho = −0.330, df = 62, p = 0.01 (B) Cohort_2010_ at week 9, Spearman’s rho = 0.767, df = 10, p = 0.021.

### 3.3 Innate Humoral Response

Mean lysozyme activity was ∼8–32× higher in Cohort_2010_ compared to Cohort_wild_ at week 5 (F = 9.9248, df = 6, 59, p<0.001). There was significant inter-annual variation (week 0, 8 & 19: Cohort_2010_ vs. Cohort_2009A_ & Cohort_2009B_ F = 3.576, df = 2, 47, p = 0.0356, but no evidence of intra-annual variation (week 0, 8 & 19: Cohort_2009A_ and Cohort_2009B_ F = 1.227, df = 1, 95, p = 0.271).

**Figure 5:**
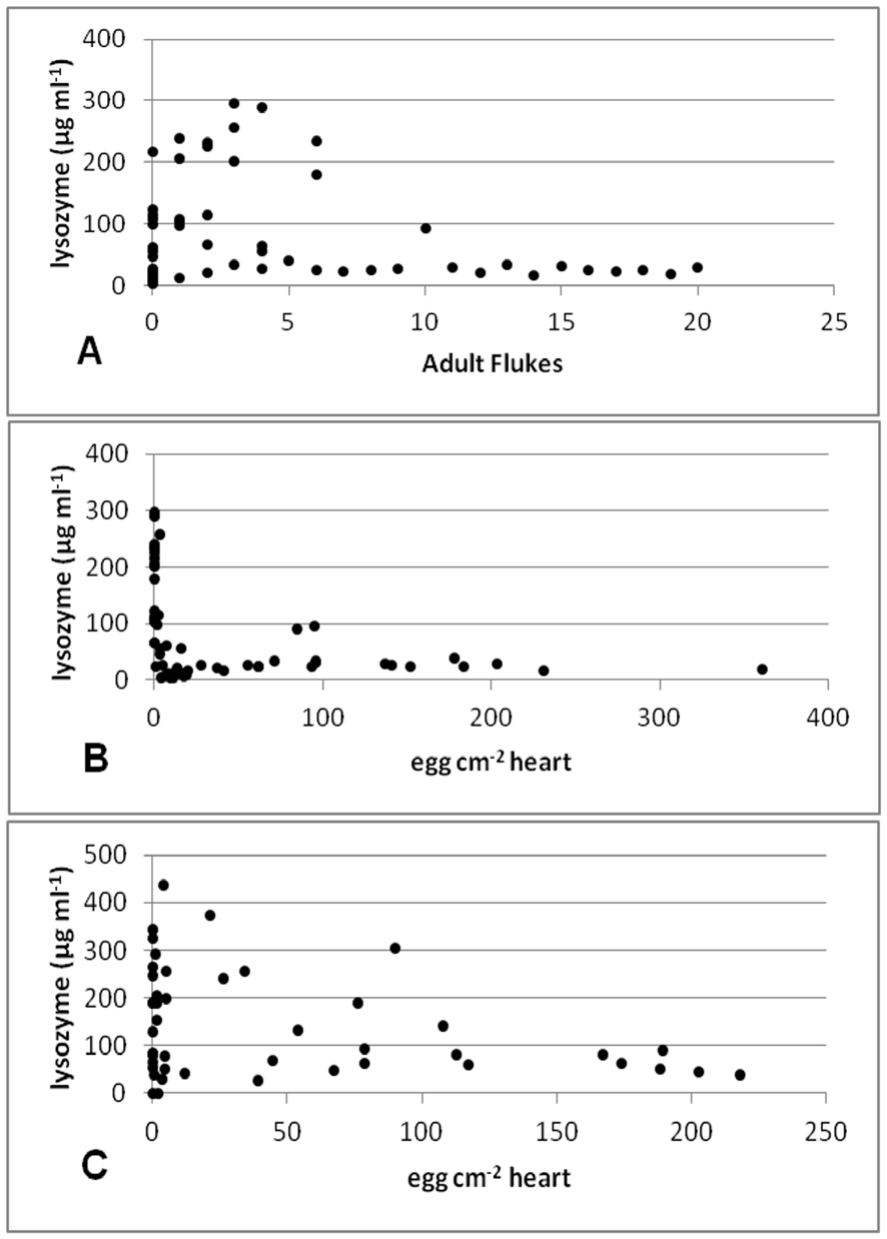
Correlation between lysozyme activity in *T. maccoyii* serum and *C. forsteri*: (A) Cohort_2009A_, Spearman’s rho = 0.2848, df = 59, p = 0.029; (B) Cohort_2009A_, Spearman’s rho = −0.2839, df = 56, p = 0.034; (C) Cohort_2010_, Spearman’s rho = −0.1261, df = 44, p = 0.042.

Early during ranching alternative complement activity (ACH50) was lower in ranched compared to wild *T. maccoyii*, but it increased over the ranching duration (Table 5). ACH50 increased 2× from Cohort_wild_ to Cohort_2010_ at week 5 of ranching (F = 4.230, df = 6, 59, p = 0.001). ACH50 decreased over ranching duration in Cohort_2009A_ (F = 33.492, df = 2, 57, p<0.001) and Cohort_2009B_ (F = 33.266, df = 1, 38, p<0.001). There was no evidence of intra-annual variation (week 8 & 19: Cohort_2009A_ vs. Cohort_2009B_ F = 0.3379, df = 1, 95, p = 0.5624) or inter-annual variation in ACH50 (week 8: Cohort_2010_ vs. Cohort_2009A_ & Cohort_2009B_ F = 0.4822, df = 2, 45, p = 0.6206).

**Figure 6:**
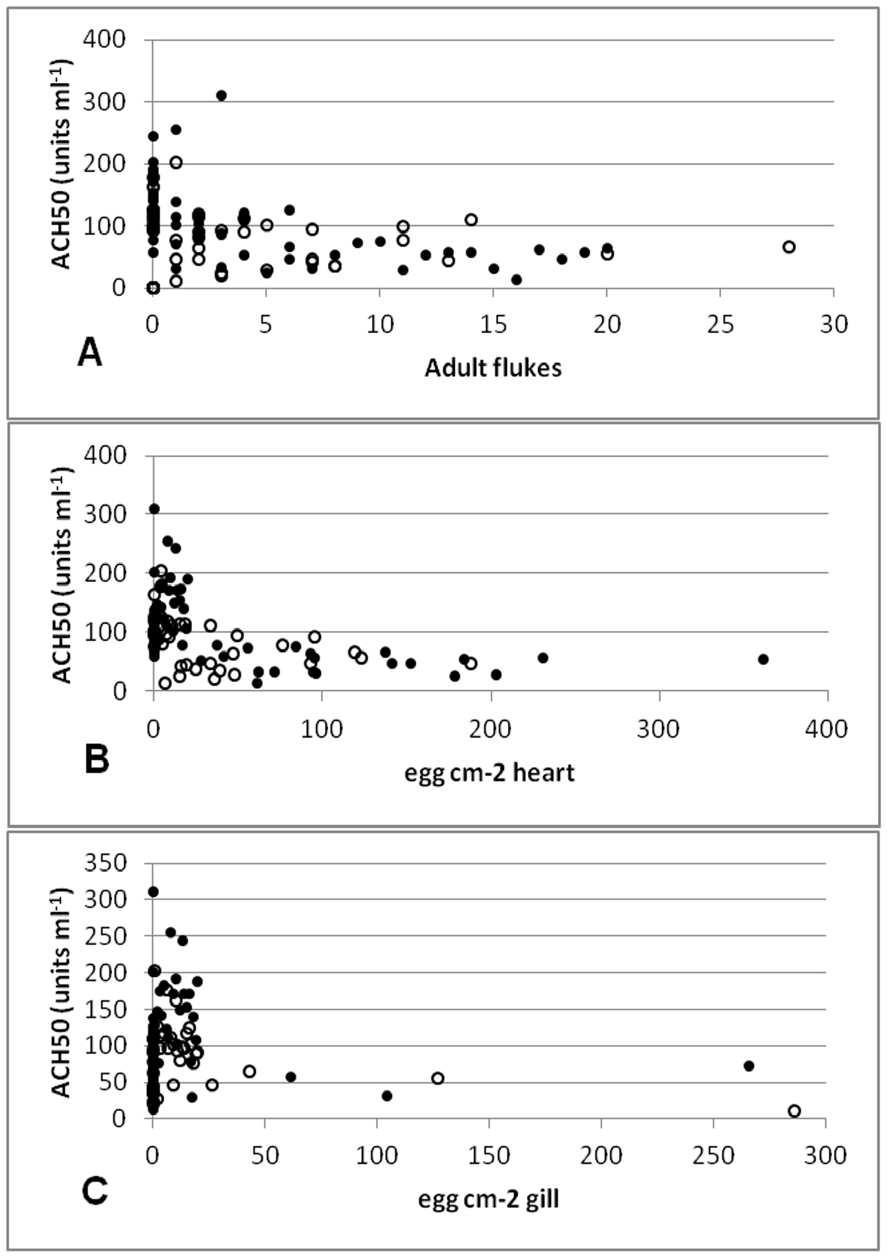
Correlations between *C. forsteri* infection and ACH50 activity in *T. maccoyii* serum from Cohort_2009A_ (•)& Cohort_2009B_ (○): (A) Cohort_2009A_ Spearman’s rho = −0.6777, df = 59, p<0.001; Cohort_2009B_ Spearman’s rho = −0.4882, df = 40, p = 0.001; (B) Cohort_2009A_ Spearman’s rho = −0.7073, df = 56, p<0.001; Cohort_2009B_ Spearman’s rho = −0.5439, df = 40, p<0.001; (C) Cohort2009A Spearman’s rho = −0.2504, df = 59, p = 0.056; Cohort_2009B_ Spearman’s rho = −0.3939. df = 30, p = 0.0311.

### 3.4 Correlations between C. forsteri Life Stages and Immune Response

There was a negative correlation between the number of adult *C. forsteri* and anti*Cardicola* antibody activity in Cohort_2008_ (Spearmans rho = −0.330, df = 62, p = 0.01), however the scatter plot shows this correlation was weak (Figure 4A). There was a positive correlation in Cohort_2010_ at week 9 of ranching between the number of eggs within the heart and anti*Cardicola* antibody activity (Spearman’s rho = 0.76667, df = 10, p = 0.02139) (Figure 4B).There were no other significant correlations in Cohort_2010_ between anti*Cardicola* antibody activity and the number of adult flukes present, the number of eggs present in the heart, and the number of eggs present in the gills. There was no correlation in Cohort_2009A_ and Cohort_2009B_ between anti*Cardicola* antibody activity, the number of adult flukes or the number of eggs present within the heart, or the number of eggs present in the gills.

In Cohort_2009A,_ there was a significant negative correlation between the number of adult flukes and lysozyme activity (Spearman’s rho = 0.2848, df = 59, p = 0.029) (Figure 5A) and between the number of eggs within the heart and lysozyme activity (Spearman’s rho = −0.2839, df = 56, p = 0.034) (Figure 5B). In Cohort_2010_, there was a significant negative correlation between lysozyme activity and eggs within the heart (Spearman’s rho = −0.1261, df = 44, p = 0.042) (Figure 5C), but no correlation with adult flukes was found. No correlations with lysozyme were found in Cohort_2009B_.

In Cohort_2009A_ and Cohort_2009B_, there was a significant negative correlation between the number of adult flukes and ACH50 activity (Cohort_2009A_: Spearman’s rho = −0.6777, df = 59, p<0.001; Cohort_2009B_: Spearman’s rho = −0.4882, df = 40, p = 0.001) (Figure 6A) and between the number of eggs within the heart and ACH50 activity (Cohort_2009A_: Spearman’s rho = −0.7073, df = 56, p<0.001; Cohort_2009B_: Spearman’s rho = −0.5439, df = 40, p<0.001) (Figure 6B). Although there was a significant correlation between the number of eggs within the gills in Cohort_2009A_ (Spearman’s rho = −0.2504, df = 59, p = 0.056) and Cohort_2009B_ with ACH50 activity (Spearman’s rho = −0.3939, df = 30, p = 0.031), this correlation was weak and based on a very few positive samples, therefore may not be accurate (Figure 6C). In Cohort_2010_, there was no correlation between ACH50 and the number of adult flukes, the number of eggs within the heart or the number of eggs within the gills.

## Discussion

The prevalence and intensity of adult and eggs of *C. forsteri* increased with ranching duration, consistent with the previous reports [1], [6]. A majority of ranched *T. maccoyii* were infected by week 5 of ranching with eggs observed within the heart from week 4. The presence of eggs at this time is consistent with the previous assumed 27 day maturation [17]. This study observed one new location of *C. forsteri* eggs infection: compacta of the heart ventricles. All eggs within this study were assumed to be *C. forsteri* due to their appearance and confirmation by qPCR [18], although eggs observed within the heart need to be further confirmed to be *C. forsteri* as previous confirmation was completed on eggs within the gills only [18]. There was a strong positive correlation between the number of adults and the number of eggs within the heart and a negative correlation between the number of eggs within the heart and eggs within the gills for all Cohorts. These correlations validate the hypothesis adults release eggs from the heart and rely on the blood stream to transport eggs to the gills [3]. Egg development was observed in the gills, but no development was observed in the heart; also consistent with previous observations [3]. The observed egg infection intensity within the heart was consistent with previous observations, estimated at ∼19000 to 1.7×106 eggs within the heart of *C. forsteri* infected *T. maccoyii* [3]. Accounting for the fish size differences, there were fewer eggs observed within the gills compared to a *Cardiola* spp. infection in Pacific Bluefin tuna, *Thunnus orientalis* (see [24]). Egg prevalence and intensity within the gills were patchy in their prevalence and intensity over the ranching duration. This observation may be related to host immune response acting against egg migration towards the gills and/or adult egg production or may be related to sampling bias due to patchy distribution of eggs within the gills [18]. Intermittent changes in the intensity and prevalence of adult flukes over the ranching season, prolonged anti*Cardicola* antibody activity and the presence of eggs within the heart and gills much longer than the maximum estimated lifespan of *C. forsteri* (see [17]), are consistent with the hypothesis *T. maccoyii* are continuously infected throughout the ranching season.

**Figure 7:**
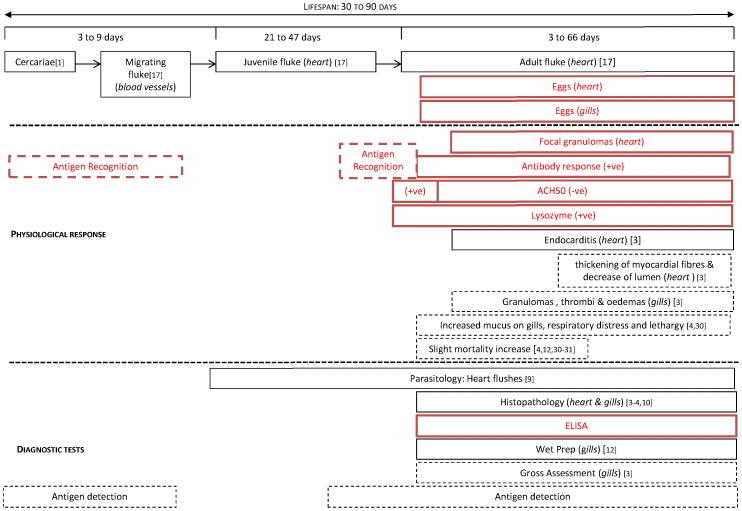
Timeline of primary *C. forsteri* infection in *T. maccoyii*: fluke life stages associated physiological response and available diagnostic tests. Items in red have been added to our knowledge from this study. Those items in dotted boxes are hypothesized but have not been experimentally proven. The order of items within physiological response and diagnostic tests sections are from most effective/prevalent to less effect/rare.

Immune response developed concurrently with *C. forsteri* infection progression, with a majority of immune changes occurring concurrent with commencing egg production. A peak in lysozyme and alternative complement activity coincides with fluke maturation. In carp infected by *S. inermis*, a similar peak and decline in complement activity was observed and believed to be related to the commencement of egg production [25]. Both lysozyme and alternative complement were negatively associated with adult and eggs at some time points, but not overall, which may suggest some parasite modulation of immune system. This hypothesis was also suggested to explain the humoral immune response in carp infected with *S. Inermis* [25]. *T. maccoyii* are ranched in the natural environment, subject to other environmental stressors which can cause changes in lysozyme and alternative complement activity during ranching [11]. Therefore, a lack of correlation with *C. forsteri* infection may also suggest limited effect of infection on lysozyme and alternative complement activity. Anti*Cardicola* antibody activity developed approximately a week after the peak in lysozyme and but a week earlier than alternative complement activity, and at a similar time as the production of eggs. Anti*Cardicola* antibody activity was present in the majority of *T. maccoyii* at approximately week 4 to 6 of ranching and remained at an elevated level for the remainder of the ranching season. This is consistent with a previously observed gradual increase in antibody response through ranching season [16]. No correlation was found between humoral immune response and eggs within the gills. It was previously believed *T. maccoyii* lysozyme activity would increase when eggs are hatching from the gills [3], although an association with immune response may rely on a larger number of eggs within the gills than found in this study. In barramundi *Lates calcarifer* infected with *Cruoricola lates*, single miracidium hatching from the gills caused little damage, but when large numbers emerged, inflammatory response and haemorrhage occurred [26]. There was a negative correlation between the number of adults and number of eggs within the gills. The significance of this observation is unknown, but may be related to the formation of granulomas trapping eggs within the heart and preventing their migration from the heart to the gills. A significant degree of intra-annual variation in infection and immune response was observed in this study. Previous studies also found annual variability in antibody levels [16] and difference between the health status of various Cohorts of *T.* maccoyii (see [21]). The relationship between the health and immune status of *T. maccoyii* prior to the commencement of ranching needs to be investigated further as it may have significant effects on infection dynamics and success of treatment. The variation also complicates utilizing the natural infection model to infer physiological and immune response to infection.

The egg stage of infection may also be responsible for a majority of the physiological response. Although previously IHC confirmed *T. maccoyii* immunoglobulins did not to bind to eggs within the heart or gills [27], this study did find a positive correlation between anti*Caridicola* activity and the number of eggs within the heart. Anti*Cardicola* antibody activity correlated with eggs in the heart at week 9 of ranching, or approximately when granulomas begin to form, but not with eggs overall. The correlation between anti*Cardicola* antibody activity and eggs at the time of granuloma development, may suggest specific antibodies play a role in *T. maccoyii* immune response to *C. forsteri* eggs. However, the number of *C. forsteri* eggs continued to increase within the heart although immune response was developed. It was previously proposed that *T. maccoyii* immune antibody response continued because of antigen from incoming *C. forsteri* larval stages [16]. This is consistent with blood fluke infections in rats (Inbred Fischer/loc male rats) (see [28]). *T. maccoyii* immunoglobulins were found to bind to *C. forsteri* marginal spines and ventral tegument in IHC [27], therefore surface antigens on adult flukes may also promote immune response. Yet there was a weak correlation between anti*Cardicola* antibody activity and number of adult flukes within this study. As adult blood flukes live in host blood, they have evolved to avoid immune response in order to ensure parasite reproduction success [29]; therefore this finding was not unexpected. More research is needed to characterize *C. forsteri* antigens and which antigens are responsible for immune response development in *T. maccoyii*.

Based on these observations and previously published information [3]–[4], [12], [17], [30]–[31] we propose the following relationships between *C. forsteri* infection events, host response, and diagnosis as shown in Figure 7. As the egg stage of infection may be the main cause of pathogenesis in *C. forsteri* infection, detection of infection at the earliest stages would aid the development of treatment protocols. Currently, infection can only be described after flukes are present in the heart and/or after eggs are produced, which delays detection to 30–50 days post infection. While there were no adult flukes detected in wild *T. maccoyii* or *T. maccoyii* at the commencement of ranching in this study, a number of fish were found with eggs and an even greater number were positive for specific antibody activity. Serial non-destructive blood sampling followed by ELISA, may be used to determine the timing infection of immature flukes invading *T. maccoyii* host, however the method would need to be validated first. Detection of anti-fluke antibody could be useful in addition to parasite detection and in some cases can have 92–97% sensitivity [32], yet in *Schistosoma* infections in humans ELISA antibody detection cannot be reliably used until three months post exposure to the parasite [33]. Alternatives, such as detection of circulating antigen may be effective for diagnosis of infection at the earliest stages. In humans with Schistosomiasis, the expression profiles of the three life stages of the parasite, egg, cercariae and adult, were determined to be different at the mRNA and protein level, and cercarial antigen has been used to diagnose in humans as early as 21 days post infection [34]. In the identification of the intermediate host of *C. forsteri*, cercariae within the intermediate host were sequenced [1]. It may be possible to use this sequence to develop a PCR blood test to detect invading cercaria in *T. maccoyii*. In addition, it may also be possible to develop a PCR to detect the changes in antigen composition at the various stages of *C. forsteri* infection. Development of non-lethal and early detection of infection is needed to monitor the infection.

In conclusion, the timeline of *C. forsteri* infection in *T. maccoyii* was validated, with confirmation of infection occurring at the initiation of ranching and continuing throughout the entire ranching duration, fluke maturation occurring at approximately 28 d post infection with eggs originating from the heart and using blood flow to migrate to the gills for development. Immune response developed concurrently with *C. forsteri* infection, with the majority of physiological response coinciding with commencing egg production. Further research is needed to confirm the origin of *C. forsteri* antigen which is responsible for immune response development and how *T. maccoyii* immune response works against infection. To aide this research, further diagnostic methods for diagnosis of infection need to be developed.
